# The Value of IGF-1 and IGFBP-1 in Patients With Heart Failure With Reduced, Mid-range, and Preserved Ejection Fraction

**DOI:** 10.3389/fcvm.2021.772105

**Published:** 2022-01-21

**Authors:** Shaohua Guo, Mengqi Gong, Gary Tse, Guangping Li, Kang-Yin Chen, Tong Liu

**Affiliations:** ^1^Tianjin Key Laboratory of Ionic-Molecular Function of Cardiovascular Disease, Department of Cardiology, Tianjin Institute of Cardiology, Second Hospital of Tianjin Medical University, Tianjin, China; ^2^Kent and Medway Medical School, Canterbury, United Kingdom; ^3^Heart Failure and Structural Heart Disease Unit, Cardiovascular Analytics Group, Hong Kong, China

**Keywords:** IGF-1, IGFBP 1, heart failure, HFrEF—heart failure with reduced ejection fraction, HFpEF—heart failure with preserved ejection fraction, HFmrEF—heart failure with mid-range ejection fraction

## Abstract

**Background:**

Previous studies have reported inconsistent results regarding the implications of deranged insulin-like growth factor 1 (IGF-1)/insulin-like growth factor-binding protein 1 (IGFBP-1) axis in patients with heart failure (HF). This study evaluates the roles of IGF1/IGFBP-1 axis in patients with HF with reduced ejection fraction (HFrEF), mid-range ejection fraction (HFmrEF), or preserved ejection fraction (HFpEF).

**Methods:**

Consecutive patients with HFrEF, HFmrEF, and HFpEF who underwent comprehensive cardiac assessment were included. The primary endpoint was the composite endpoint of all-cause death and HF rehospitalization at one year.

**Results:**

A total of 151 patients with HF (HFrEF: *n* = 51; HFmrEF: *n* = 30; HFpEF: *n* = 70) and 50 control subjects were included. The concentrations of IGFBP-1 (*p* < 0.001) and IGFBP-1/IGF-1 ratio (*p* < 0.001) were significantly lower in patients with HF compared to controls and can readily distinguish patients with and without HF (IGFBP-1: areas under the curve (AUC): 0.725, *p* < 0.001; IGFBP-1/IGF-1 ratio: AUC:0.755, *p* < 0.001; respectively). The concentrations of IGF-1, IGFBP-1, and IGFBP-1/IGF-1 ratio were similar among HFpEF, HFmrEF, and HFrEF patients. IGFBP-1 and IGFBP-1/IGF-1 ratio positively correlated with N-terminal probrain natriuretic peptide (NT-proBNP) levels (*r* = 0.255, *p* = 0.002; *r* = 0.224, *p* = 0.007, respectively). IGF-1, IGFBP-1, and IGFBP-1/IGF-1 ratio did not predict the primary endpoint at 1 year for the whole patients with HF and HF subtypes on both univariable and multivariable Cox regression.

**Conclusion:**

The concentrations of plasma IGFBP-1 and IGFBP-1/IGF-1 ratio can distinguish patients with and without HF. In HF, IGFBP-1 and IGFBP-1/IGF-1 ratio positively correlated with NT-proBNP levels.

## Introduction

Heart failure (HF) is the final common pathway of many cardiovascular diseases and can be classified into reduced ejection fraction (HFrEF), mid-range ejection fraction (HFmrEF), or preserved ejection fraction (HFpEF) based on the 2016 European Society of Cardiology (ESC) guideline for HF (1). Risk stratification should be based on a multimodality approach but can differ between HF subtypes (2–5). Although survival for patients with HFrEF has been improved substantially due to advances in drug and device-based therapies, the use of medications to improve prognosis in patients with HFmrEF and HFpEF is less well defined. Patients with HFmrEF and HFpEF constitute more than one-half of the HF cohort, but the risk stratification for both subtypes remains difficult (6–9). Nevertheless symptomatic HFmrEF and HFpEF show a poorer prognosis compared to their HFrEF counterparts (10). Better understanding the pathophysiology of the three HF subtypes will provide additional insights for guiding medical therapies (11).

Circulating biomarkers reflect the pathophysiological state of HF and are of potential value for its diagnosis and prognosis (12, 13). The peptic hormone-insulin-like growth factor 1(IGF-1) regulates proliferation, differentiation, metabolism, and cell survival in various tissues. Over recent years, an increasing number of studies have reported the link of IGF-with to all-cause mortality and cardiovascular diseases, such as HF, atrial fibrillation, and stroke (14–17). By upregulating the IGF1-PI3K-Akt pathway, IGF-1 tends to show cardioprotective effects (18), improves cardiomyopathy (19), and modulates the cellular processes implicated in short-term ventricular remodeling of the infarcted myocardium (20). IGF-binding proteins bind to IGF-1, thereby regulating its activity. Among these IGF-binding proteins, in particular, IGF-binding protein 1 (IGFBP-1) has numerous actions, including peripheral binding and potent inhibition of IGF-1 (21). A previous study (22) has investigated the ability of IGF-1/IGFBP-1 to distinguish between HF subtypes, and found that IGF-1 levels were different between HFpEF and HFrEF, and have prognostic roles. In this study, we investigated the plasma concentrations of IGF-1 and IGFBP-1 in patients with HF and compared their levels between HF subtypes, their correlations with N-terminal prohormone brain natriuretic peptide (NT-proBNP), and their prognostic values.

## Methods

### Study Population

This study enrolled consecutive patients from October 2018 to January 2020. The inclusion criteria were: (1) HF symptoms or signs; (2) NT-proBNP >125 ng/ml; and (3) patients were divided into left ventricular ejection fraction (LVEF) < 40% (HFrEF); LVEF ≥ 40% and < 50% (HFmrEF) and LVEF ≥ 50% (HFpEF) groups. The control group enrolled patients referred for elective angiography or treatment of uncontrolled hypertension with NT-proBNP ≤ 125 ng/l. Exclusion criteria included acute myocardial infarction, myocarditis, moderate-to-severe valvular heart disease, severe systemic inflammatory disease, or severe renal or hepatic disease. The study was approved by the local ethics committee of the Second Hospital of Tianjin Medical University and conformed to the Declaration of Helsinki.

### Clinical, Biochemical, and Echocardiographic Data

Baseline data with regard to demographic and clinical variables involving age, gender, hospital stay, smoking history, comorbidities (such as hypertension, diabetes, coronary revascularization history, and atrial fibrillation), blood pressure, heart rate, biochemical results (in particular, NT-proBNP), and discharge medication were collected.

Blood samples were drawn at rest and collected with ethylenediamine tetraacetic acid (EDTA) anticoagulant tubes to analyze routine laboratory parameters. The blood tubes were centrifuged at 3,000 *g* at room temperature for 10 min and plasma was separated from cellular compartments and stored at −80°C for later analysis of IGF-1 and IGFBP-1. An ELISA was performed to measure the concentration of IGF-1 and IGFBP1 using IGF-1 and IGFBP1 assay kit (Cusabio, China).

Echocardiography was performed using a standard ultrasound system (PHILIPS iE33). LVEF was measured based on modified biplane Simpson's method. Measurement of left atrial anterior and posterior diameter (LAD), interventricular septum thickness (IVS), left ventricular end-diastolic diameter, and left ventricular end-systolic diameter were from parasternal long-axis view.

### Follow-Up and Outcomes

All the patients with HF continued the standardized treatment for HF after discharge. Patients were followed up by clinical visits or telephone calls for 12 months. The primary endpoint was the composite endpoint of all-cause death and HF rehospitalization at 1 year. The follow-up time was calculated from discharge to all-cause death, first readmission, or termination of the study.

### Statistics

Baseline continuous variables were reported as mean ± SD or median and interquartile range, which is based on a continuous distribution of data: Student's *t*-test or ANOVA is used for normal distribution, and the Mann–Whitney test or the Kruskal–Wallis test is used for abnormal distribution. Categorical variables are expressed as numbers and percentages and compared using the Chi-square test or Fisher's exact test.

Concentrations of IGF-1, IGFBP-1, and IGFBP-1/IGF-1 ratio were compared in HFrEF, HFmrEF, HFpEF, and controls. The correlation was performed between levels of IGF-1, IGFBP-1, IGFBP-1/ IGF-1 ratio, and NT-proBNP using Pearson's *r*. The diagnostic value of IGF-1, IGFBP-1, and IGFBP-1/ IGF-1 ratio to identify HF were investigated and compared *via* the areas under the curve (AUCs) of receiver operating characteristics (ROC) curves. The Cox proportional hazard model was also performed to investigate the prognostic value of IGF-1 concentration, IGFBP-1 concentration, and IGFBP-1/IGF-1 ratio. Log-rank tests for the Kaplan–Meier survival curves were performed according to different HF subtypes. All data were analyzed using SPSS statistical software (SPSS 25.0) R programming version 4.1.1. A *p*-value < 0.05 was considered statistically significant.

## Results

### Baseline Characteristics and Biomarkers of Patients With HF and Controls

**Figure 1:**
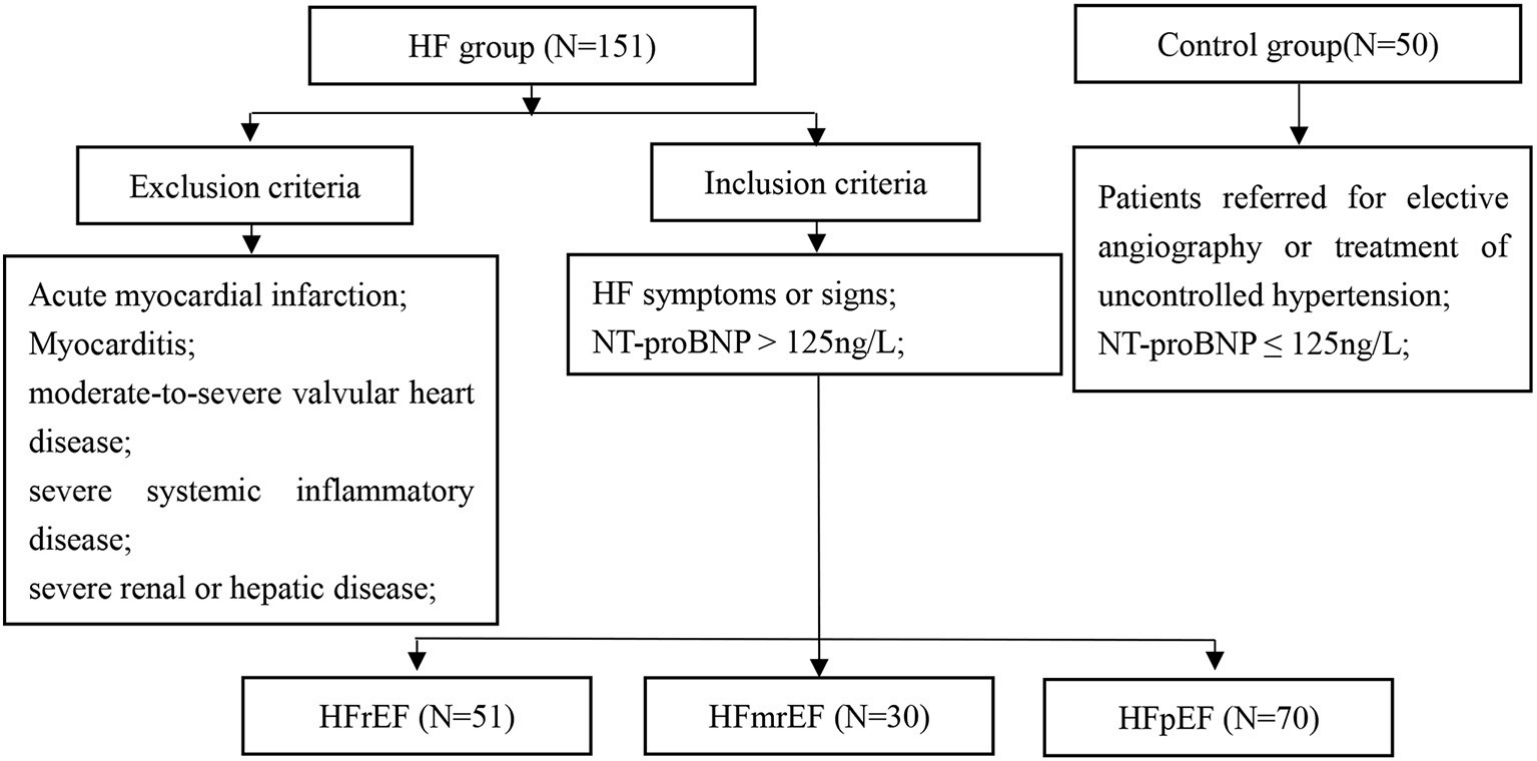
Study flowchart. HF, heart failure; HFrEF, HF with reduced ejection fraction; HFmrEF, HF with mid-range ejection fraction; HFpEF, HF with preserved ejection fraction; NT-proBNP, N-terminal pro-B natriuretic peptide.

A total of 163 patients with HF were enrolled. Of these, 12 patients were lost to follow-up, and therefore 151 consecutive patients with HF (mean age 68.9 ± 11.4 years; 59.6% men) were included in the final analysis. In total, 50 subjects without HF were included as controls (Figure 1). The baseline characteristics of the study cohort are shown in Table 1. Compared to controls, patients with HF had a higher male frequency (59.6 vs. 42.0%, *p* = 0.030), had a longer hospital stay, had higher rates of atrial fibrillation, prior myocardial infarction, stroke, prior coronary revascularization, and more likely to use digoxin, diuretics, and cardioprotective medicine at discharge, such as beta-blocker, spironolactone, angiotensin system antagonist [angiotensin-converting enzyme inhibitor (ACEI), angiotensin receptor blocker (ARB), and angiotensin receptor-neprilysin inhibitors (ARNI)]. In addition, patients with HF had a larger atrium (*p* < 0.001) and ventricle (*p* < 0.001), and lower LVEF (*p* < 0.001) compared to controls.

**Table 1:** Baseline characteristics of study participants.

	**HF (*n* = 151)**	**Control (*n* = 50)**	***P* value**
Demographics and vital signs			
Age (years)	71.0 (61.8, 77.0)	66.0 (60.3, 72.0)	0.048
Male	90 (59.6%)	21 (42.0%)	0.030
Hospital stay (days)	7.5 (5.7, 11)	4 (3, 5.7)	<0.001
Systolic BP (mmHg)	130.5 (114.0, 150.0)	141.5 (126.5, 149.0)	0.025
Diastolic BP (mmHg)	80.0 (69.7, 89.2)	81.0 (72.2, 86.0)	0.872
Heart rate (bpm)	80.0 (69.7, 82.0)	70.5 (63.0, 80.5)	0.002
Medical history			
Atrial fibrillation	56 (37.1%)	3 (6%)	<0.001
Prior MI	52 (34.4%)	3 (6.0%)	<0.001
Hypertension	108 (71.7%)	38 (76.0%)	0.689
Diabetes mellitus	62 (41.3%)	13 (26.0%)	0.052
Stroke	44 (29.1%)	5 (10.0%)	0.006
COPD	8 (5.3%)	0 (0.0%)	0.214
Coronary revascularization history	45 (29.8%)	6 (12.0%)	0.012
Smoking history	67 (44.4%)	18 (36.0%)	0.299
Echocardiographic parameters			
LVEF, %	48.0 (38.0, 59.2)	64.00 (60.0, 67.7)	<0.001
LAD (mm)	44.5 (40.3, 50.0)	38.2 (35.0, 40.6)	<0.001
LVEDD (mm)	51.9 (47.8, 58.8)	47.9 (44.1, 50.5)	<0.001
IVS (mm)	9.35 (8.4, 11.0)	9.00 (8.40, 9.60)	<0.001
Discharge medications			
ARB or ACEI or ARNI	97 (64.2%)	22 (44.9%)	0.017
Digoxin	18 (11.9%)	0 (0.0%)	0.011
Beta blocker	96 (63.6%)	22 (44.9%)	0.021
Calcium channel blocker	48 (31.8%)	20 (40.8%)	0.246
Spironolactone	87 (57.6%)	3 (6.1%)	<0.001
Diuretics	89 (59.3%)	5 (10.2%)	<0.001
Biomarkers			
Creatinine (umol/L)	88.8 (68.7, 126.4)	73.3 (60.3, 87.6)	<0.001
Hemoglobin (g/L)	130 (108, 144)	136 (128, 143)	0.036
Troponin I (ng/ml)	0.02 (0.01, 0.10)	0.02 (0.01, 0.03)	0.521
CK-MB (U/L)	14.0 (10.0, 22.0)	11.2 (9.5, 19.2)	0.181
D-dimer (mg/L)	797.9 (439.1, 1,208.1)	328.3 (233.2, 510.6)	<0.001
Total cholesterol (mmol/L)	4.1 (3.3, 5.3)	4.6 (3.7, 5.1)	0.241
Triglyceride (mmol/L)	1.1 (0.8, 1.6)	1.7 (1.1, 2.4)	<0.001
NT-proBNP (ng/L)	3,334.5 (1,855.7, 8,112.2)	86.7 (38.9, 210.0)	<0.001
IGF-1 (ng/ml)	50.9 (37.4, 72.6)	50.1 (34.8, 67.3)	0.392
IGFBP-1 (ng/ml)	60.3 (5.7, 461.4)	439.7 (404.2, 523.2)	<0.001
IGFBP-1/IGF-1	1.36 (0.1, 8.7)	8.5 (6.3, 13.1)	<0.001

*ACEI, angiotensin-converting enzyme inhibitor; ARB, angiotensin receptor blocker; ARNI, angiotensin receptor-neprilysin inhibitors; BP, blood presure; COPD, chronic obstructive pulmonary disease; HF, heart failure; IGF-1, insulin-like growth factor 1; IGFBP-1, IGF binding protein 1; IVS, interventricular septum thickness; LAD,left atrial anterior and posterior diameter; LVEDD, left ventricular end-diastolic diameter; LVEF, left ventricular ejection fraction; MI, myocardial infaction; NT-proBNP, N-terminal prohormone brain natriuretic peptide*.

Heart failure had a higher creatinine level (*p* < 0.001) and a lower hemoglobin level (*p* = 0.036) compared with control. NT-proBNP levels were higher in HF than in control subjects without HF (*p* < 0.001). The levels of IGF-1 in patients with HF and controls were, median (IQR), 50.9 (37.4, 72.6) ng/ml, and 50.0 (34.8, 67.3) ng/ml, respectively, but no difference was found between the two groups (*p* = 0.392). In contrast, the levels of IGFBP-1 (*p* < 0.001) and IGFBP-1/IGF-1 ratio (*p* < 0.001) were significantly lower in patients with HF compared with controls.

**Figure 2:**
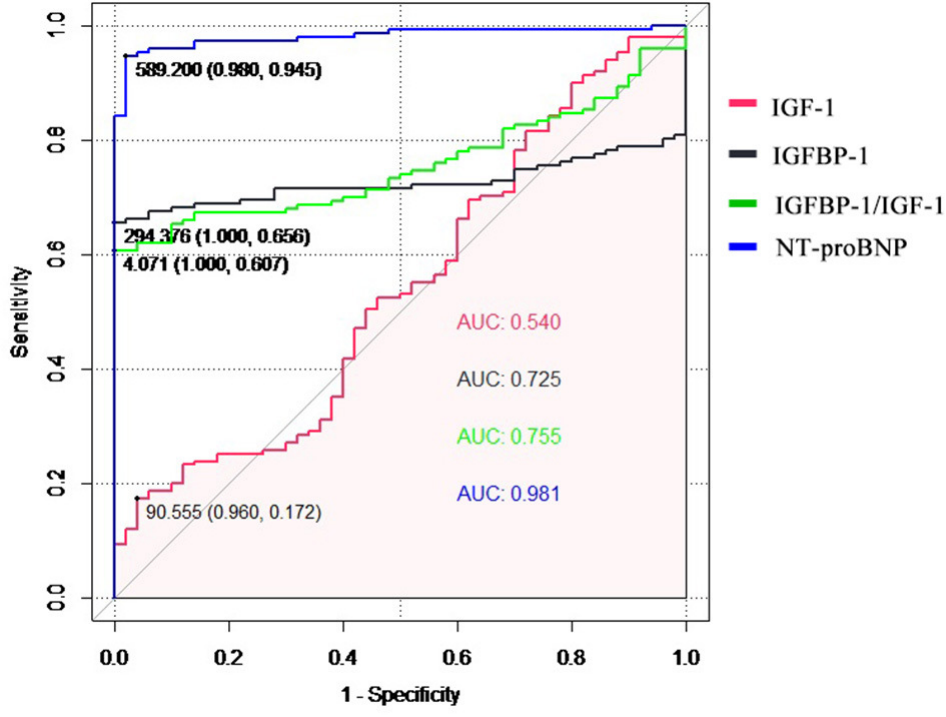
The receiver operating characteristic (ROC) curve of the diagnostic ability for heart failure of IGF-1, IGFBP-1, IGFBP-1/IGF-1 ratio, and NT-proBNP. IGF-1, insulin-like growth factor 1; IGFBP-1, IGF binding protein 1; NT-proBNP, N-terminal pro-B natriuretic peptide.

The diagnostic performance for HF diagnosis was analyzed by ROC analysis for IGF-1, IGFBP-1, and the IGFBP-1/IGF-1 ratio in the patients with HF and controls (Figure 2). IGFBP-1 and IGFBP-1/IGF-1 ratios have moderate values for distinguishing between patients with HF and non-HF (AUC = 0.725 and 0.755, respectively). IGF-1 was not useful for this classification. The predictive abilities of NT-proBNP were superior to those of IGFBP-1 and IGFBP-1/IGF-1 ratio (AUC for NT-proBNP, 0.981).

### Baseline Characteristics and Biomarkers in HFrEF, HFmrEF, and HFpEF

Of the 151 patients with HF, 51 had HFrEF, 30 had HFmrEF, and 70 had HFpEF. Their baseline characteristics are summarized in Table 2. As compared with HFpEF and HFmrEF, patients with HFrEF were more commonly men, had lower systolic blood pressure, tended to have a prior myocardial infarction, and were more likely to be prescribed with angiotensin system antagonist (ACEI, ARB, and ARNI), diuretic, spironolactone, and digoxin. Compared to HFrEF and HFmrEF, patients with HFpEF were less likely to have an ischemic etiology of HF (*p* = 0031). Otherwise, according to echocardiography, patients with HFrEF had more enormous left atrium and left ventricle and thinner IVS than patients with HFpEF and HFmrEF. In contrast, the percent of calcium channel blockers was higher in the HFpEF group than in HFrEF and HFmrEF group. There was no difference in the primary endpoint among the three groups (HFrEF 58.5% vs. HFmrEF 43.3% vs. HFpEF 51.4%, (*p* = 0.395), however, HFrEF was a trend to a higher risk of all-cause death at 12 months compared with HFmrEF and HfpEF (*p* = 0.069).

**Table 2:** Baseline characteristics of heart failure patients.

	**HFrEF (*N* = 51)**	**HFmrEF (*N* = 30)**	**HFpEF (*N* = 70)**	***P* value**
Demographics and vital signs				
Age (years)	68.4 ± 11.2	66.3 ± 11.9	70.3 ± 11.1	0.255
Male	41 (80.4%)	19 (63.3%)	30 (42.9%)	<0.001
Hospital stay (days)	9.6 ± 5.7	7.7 ± 3.8	9.2 ± 5.8	0.316
Systolic BP (mmHg)	123.8 ± 26.6	139.7 ± 28.7	137.9 ± 27.1	0.008
Diastolic BP (mmHg)	80.5 ± 16.8	87.3 ± 19.4	77.2 ± 15.0	0.022
Heart rate (bpm)	85.4 ± 20.8	81.6 ± 14.1	78.2 ± 20.2	0.135
NYHA I	2 (3.9%)	6 (20.0%)	5 (7.1%)	0.305
NYHA II	13 (25.5%)	8 (26.7%)	18 (25.7%)	
NYHA III	25 (49.0%)	13 (43.3%)	32 (45.7%)	
NYHA IV	11 (21.6%)	3 (10.0%)	5 (21.4%)	
Medical history				
Ischemic etiology	34 (66.7%)	22 (73.3%)	34 (48.6%)	0.031
Atrial fibrillation	14 (27.5%)	9 (30.0%)	33 (47.1%)	0.119
Prior MI	29 (56.9%)	9 (30.0%)	14 (20.0%)	<0.001
Hypertension	31 (60.8%)	22 (73.3%)	55 (78.6%)	0.193
Diabetes mellitus	25 (49.0%)	16 (53.3%)	21 (30.4%)	0.041
Stroke	15 (29.4%)	5 (16.7%)	24 (34.3%)	0.206
COPD	2 (3.9%)	3 (10.0%)	3 (4.3%)	0.437
Coronary revascularization history	18 (35.2%)	5 (16.7%)	22 (31.4%)	0.192
Smoking history	26 (51.0%)	13 (43.3%)	28 (40.0%)	0.483
Echocardiographic parameters				
LVEF, %	30.0 (24.0, 37.7)	41.0 (41.00, 47.00)	60.0 (55.2, 63.7)	<0.001
LAD (mm)	48.7 ± 9.0	43.4 ± 6.8	45.2 ± 7.0	0.008
LVEDD (mm)	59.3 ± 11.1	54.2 ± 7.8	48.3 ± 8.4	<0.001
IVS (mm)	8.7 ± 2.1	10.0 ± 1.9	10.4 ± 2.3	0.001
Discharge medications				
ARB or ACEI or ARNI	43 (84.3%)	18 (60.0%)	36 (51.4%)	0.001
Digoxin	13 (25.5%)	1 (3.3%)	4 (5.7%)	0.001
Beta blocker	36 (70.6%)	19 (63.3%)	41 (58.6%)	0.398
Calcium channel blocker	8 (15.7%)	8 (26.7%)	32 (45.7%)	0.002
Spironolactone	41 (80.4%)	17 (56.7%)	29 (41.4%)	<0.001
Diuretic	41 (80.4%)	16 (53.3%)	32 (46.4%)	0.001
Biomarkers				
Creatinine (umol/L)	99.7 (79.7, 123.9)	79.1 (62.8, 121.5)	88.5 (65.5, 147.8)	0.206
Hemoglobin (g/L)	130.0 (117.0, 146.5)	130.5 (113.0, 147.0)	128.0 (103.0, 141.0)	0.448
Troponin I (ng/ml)	0.05 (0.01, 0.11)	0.05 (0.01, 0.65)	0.01 (0.00, 0.06)	0.087
CK-MB (U/L)	14.0 (11.5, 23.5)	17.3 (11.0, 28.1)	13.9 (9.0, 23.6)	0.304
D-dimer (mg/L)	879.5 (539.38, 1,554.1)	591.2 (392.4, 1,039.6)	619.1 (397.6, 2,473.6)	0.167
Total cholesterol (mmol/L)	3.7 (2.9, 4.6)	4.4 (3.5, 5.6)	4.5 (3.7, 5.6)	0.008
Triglyceride (mmol/L)	0.9 (0.6, 1.5)	1.2 (0.8, 1.6)	1.3 (0.9, 1.8)	0.235
NT-proBNP (ng/L)	5,981.5 (2,349.7, 11,501.0)	3,550.0 (2,495.0, 7,813.0)	2,488.0 (1,633.7, 5,216.0)	0.014
IGF-1 (ng/ml)	49.4 (36.0, 73.5)	50.3 (33.6, 88.7)	51.3 (39.2, 62.4)	0.979
IGFBP-1 (ng/ml)	103.3 (6.1, 615.8)	133.3 (13.2, 497.9)	50.9 (4.7, 487.3)	0.456
IGFBP-1/IGF-1	2.3 (0.1, 9.7)	1.5 (0.3, 12.3)	1.2 (0.1, 8.7)	0.617
Outcomes				
Primary endpoint, n (%)	30 (58.5%)	13 (43.3%)	36 (51.4%)	0.395
Heart failure hospitalization, n (%)	21 (41.2%)	11 (36.7%)	29 (41.4%)	0.890
All cause death, n (%)	9 (17.6%)	2 (6.7%)	7 (10.0%)	0.269

*HFrEF, heart failure with reduced ejection fraction; HFmrEF: heart failure with mid-range ejection fraction; HFpEF: heart failure with preserved ejection fraction; NYHA, New York Heart Association. Other abbreviations are as Table 1*.

**Figure 3:**
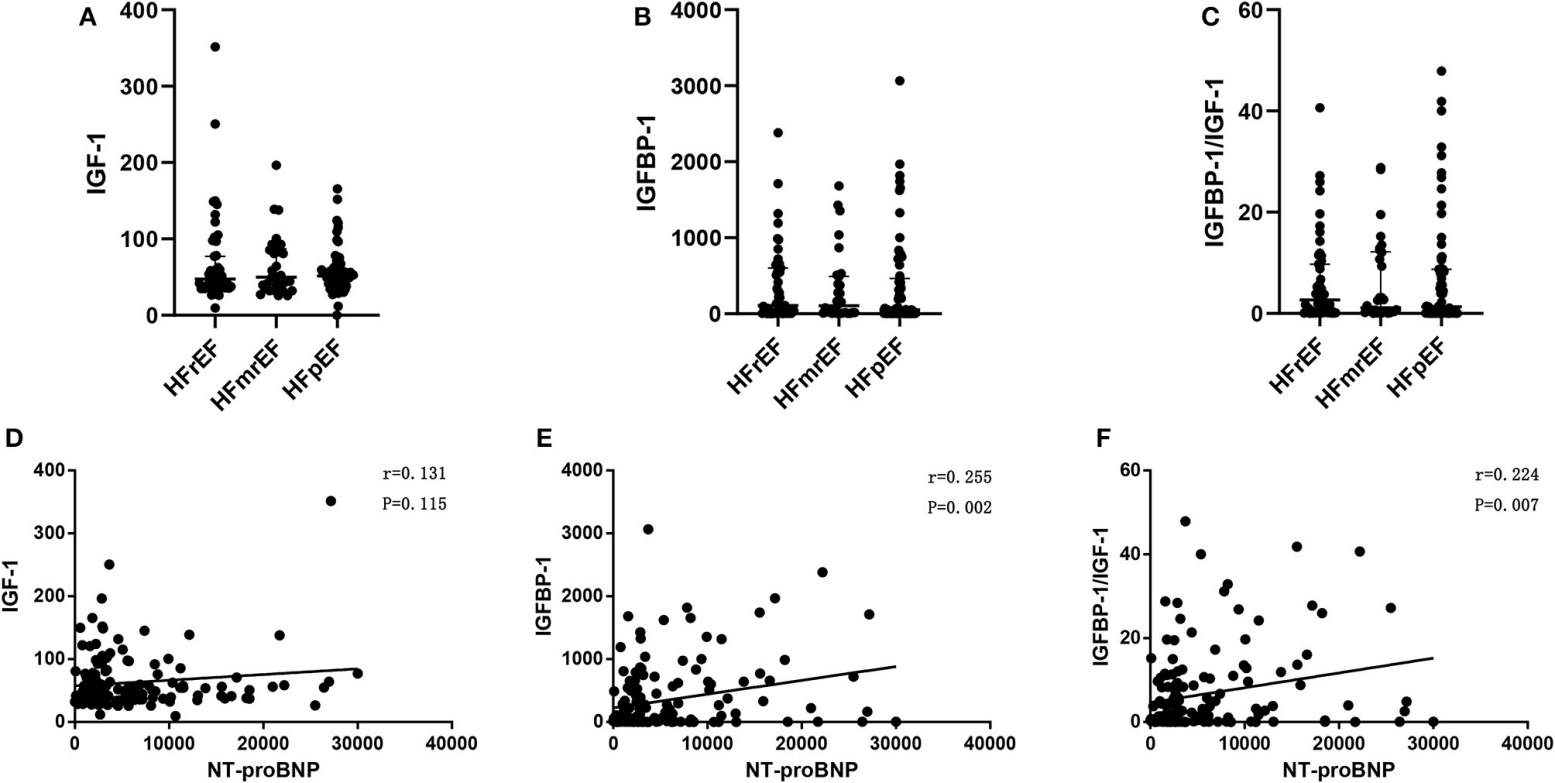
IGF-1 **(A)**, IGFBP-1 **(B)**, and IGFBP-1/IGF-1 ratio **(C)** in HFrEF, HFmrEF,and HFpEF; Pearson's correlation between IGF-1 and NT-proBNP **(D)**, IGFBP-1 and NT-proBNP **(E)**, and IGFBP-1/IGF-1 ratio and NT-proBNP **(F)**, in patients with heart failure. Abbreviations as Figures 1, 2.

The levels of IGF-1, IGFBP-1, and IGFBP-1/IGF-1 ratio were similar among patients with HFpEF, HFmrEF, HFrEF (Table 2 and Figures 3A–C). The difference of these biomarkers between HFrEF, HFmrEF, and HFpEF remained insignificant after adjustment for gender, age, and NT-proBNP. There was a progressive increase in NT-proBNP levels from HFpEF to HFmrEF to HFrEF, with patients with HFrEF having the highest levels (*p* = 0.014, Table 2). IGFBP-1 levels and IGFBP-1/IGF-1 ratio were positively correlated with NT-proBNP levels (*r* = 0.255, *p* = 0.002; *r* = 0.224, *p* = 0.007, respectively; Figures 3D–F).

**Figure 4:**
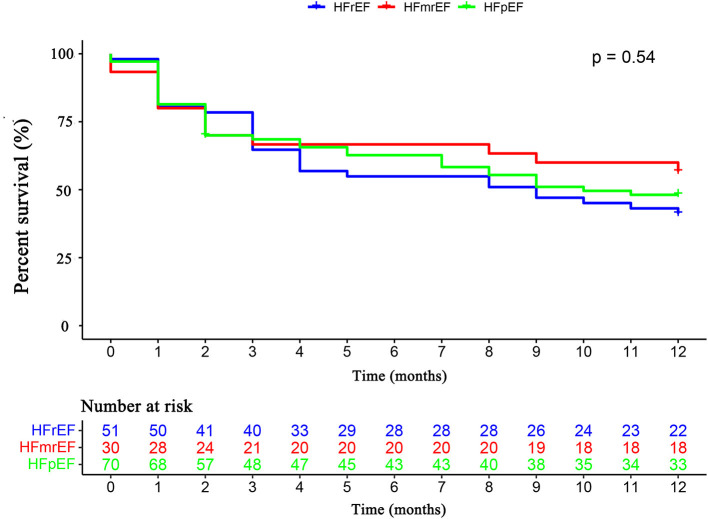
The Kaplan–Meier survival curves according to HFrEF, HFmrEF, and HFpEF. Abbreviations as for Figure 1.

**Figure 5:**
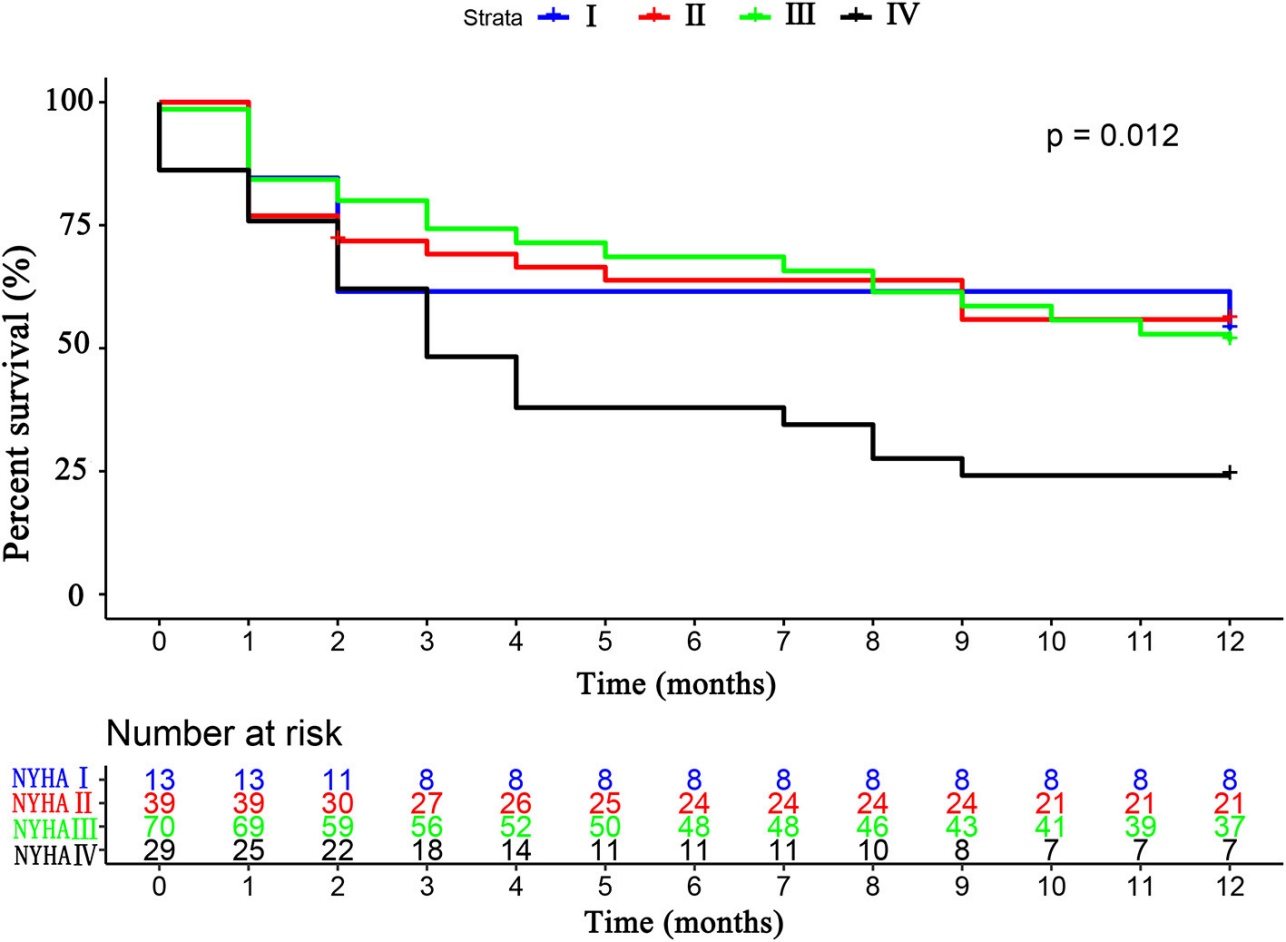
The Kaplan–Meier survival curves according to the NYHA cardiac classification. NYHA, New York Heart Association.

**Figure 6:**
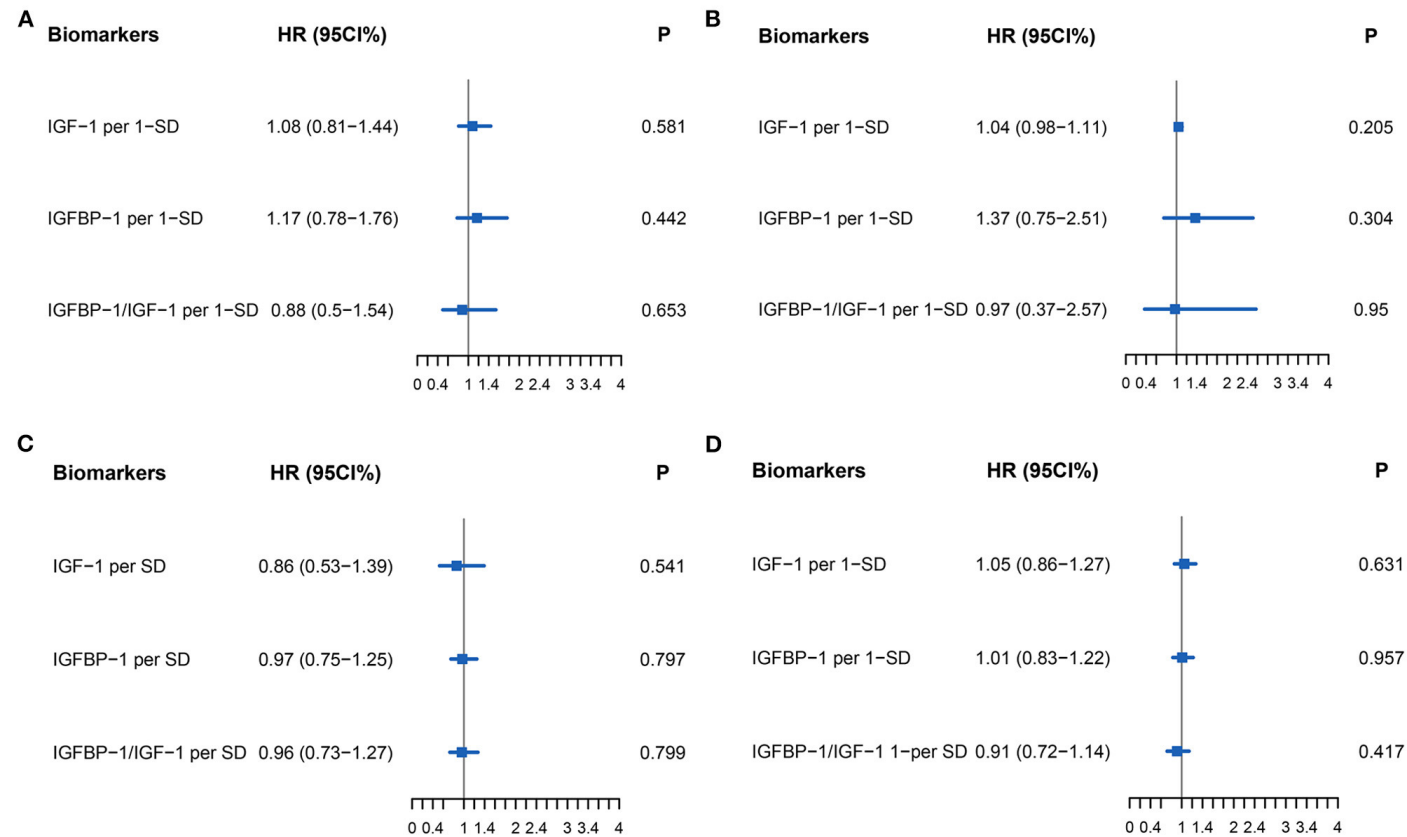
Forest plot of biomarkers IGF-1 (Per 1-SD), IGFBP-1 (Per 1-SD), and IGFBP-1/IGF-1 (Per-SD) for Cox multivariable adjustment of age, gender, atrial fibrillation, and NT-proBNP for the prediction of the primary endpoint (all-cause death and rehospitalization for heart failure at one-year follow-up) in patients with heart failure. **(A)** for HFrEF, **(B)** for HFmrEF, **(C)** for HFpEF, **(D)** for all patients with HF. Abbreviations as for Figures 1, 2.

The primary endpoint occurred in 79 (52.3%) patients, of whom 16 (10.6%) died and 63 (41.7%) were rehospitalized for HF. There was no difference in the primary endpoint among the three groups (HFrEF 58.5% vs. HFmrEF 43.3% vs. HFpEF 51.4%, *p* = 0.540, Figure 4). As shown in Figure 5, with the aggravation of cardiac function (New York Heart Association, NYHA), the incidence of the primary endpoint was significantly increased (*p* = 0.012). Multivariable Cox regression showed that IGF-1 levels, IGFBP-1 levels, and IGFBP-1/IGF-1 ratio were not predictive of prognosis after adjusting for age, gender, atrial fibrillation, and NT-proBNP in patients with HF (Figure 6).

## Discussion

This is, to the best of our knowledge, the first study comparing the concentrations of IGF-1 between various HF subtypes. The main results are that (1) IGFBP-1 and IGFBP-1/IGF-1 ratio were significantly lower in patients with HF compared to those without HF, (2) in HF, IGF-1, IGFBP-1, and IGFBP-1/IGF-1 ratio did not differ among HFpEF, HFmrEF, and HFrEF, and (3) IGFBP-1 and IGFBP-1/IGF-1 ratio positively correlated with NT-proBNP.

There is an increasing body of evidence that IGF-1 can have protective roles in the heart. Patients with HF are more likely to have a lower concentration of IGF-1. As supported by previous studies, ACEIs have been shown to improve survival in patients with HFrEF (23) and are recommended for the treatment of every patient with HFrEF according to guidelines (1), and can regulate IGF-1 levels (24, 25). Nevertheless, studies on the relationship between IGF-1concentrations and cardiovascular disease vary significantly, reporting to be reduced, normal, and even increased. On the one hand, lower IGF-1 levels seemed to be harmful and associated with diastolic dysfunction and even HFpEF (26). On the other hand, as shown by Faxen et al. (26) compared with the control group, IGF-1 levels were higher in HFpEF but lower in HFrEF. The normal range of IGF-1 in patients with HF was also ever reported (27). Our patients were enrolled consecutively in the hospital, and all samples were obtained during the acute phase. However, IGF-1 could neither identify patients with HF from controls nor distinguish HF subtypes. We supposed that IGF-1 might have a protective role in the process of modulating heart activity, and as a possibility, the divergent results may be due to the susceptibility of IGF-1 to baseline environments, such as age, race, and acute period and unrecognized differences in lifestyle factors modulating IGF-1 levels.

Insulin-like growth factor-binding proteins are widely expressed in most tissues, and are endocrine and autocrine/paracrine regulators of IGF activity, which is essential for this crucial physiological system. IGF-1 activity is regulated by IGFBPs. However, IGFBPs function their biological roles not only by binding to IGF but also play roles independent of the IGF system (28, 29). IGFBP-1 binds IGF1 and IGF2 with equal affinity, inhibiting or enhancing IGF actions (30, 31). Previous studies reported diverse conclusions about the prognostic role of IGFBP-1. One study showed that IGFBP-1 was associated with long-term all-cause and cancer mortality but not cardiovascular events (32). Other studies indicated that IGFBP-1 is a long-term predictor of HF in survivors of a first acute myocardial infarction (33) and predicts adverse clinical outcomes during outpatient follow-up of patients with chronic HF (34). In contrast, consistent with our results, Faxen et al. reported IGFBP-1 was similar in HFpEF and HFrEF phenotypes and revealed no associations with outcomes (22).

In this study, lower IGFBP-1 concentration and IGFBP-1/IGF-1 ratio values showed a correlation from controls to patients with HF, while neither IGF-1 nor IGFBP-1 have value in distinguishing HF subtypes or predicting prognosis. In addition, the correlation between levels of IGFBP-1 and IGFBP-1/IGF-1 ratio and NT-proBNP, a well-recognized prognostic marker and indicator of elevated ventricular filling pressures among patients regardless of ejection fraction (35, 36), indicated that IGFBP-1 and IGFBP1/IGF-1 may serve as a supplementary to better estimate prognosis of HF, despite their negative role in this study.

Heart failure with reduced ejection fraction, HFmrEF, and HFpEF sharing common clinical features constitute different entities with distinct pathogenetic backgrounds. Efforts are made to find biomarkers identifying subjects with different HF entities. Recent years have emerged studies reporting several biomarkers which can discriminate HFpEF from HFrEF. High-Density Lipoprotein Particle Subfractions can distinguish between HFpEF and HFrEF (37). A study investigating inflammation mediated by the tumor necrosis factor-alpha (TNFa) axis in patients with HF indicated that there was a significant difference in TNF receptor-2 (TNFR2) between patients with HFrEF and HFpEF (38). An ensemble of the male-specific transcriptomic panel with NT-proBNP has been estimated to be able to differentiate between HFpEF and HFrEF (39). Otherwise, some biomarkers cannot distinguish HF subtypes but have an indicative value. For instance, cystatin C was higher in HFpEF than HFrEF but not significantly (40). Higher levels of adiponectin were associated with the adverse outcome only in HFrEF, not HFpEF (41). Growth differentiation factor 15 is similarly elevated and has an independent prognostic utility in both HFrEF and HFpEF (42), without differentiating value.

Several limitations of this study must be acknowledged. First, the definitive determination of cause and effect relationships was not clear due to the retrospective observational nature of the present study. As reported by previous studies, the activity of IGF-1 and IGFBP-1 are regulated by insulin, while it is a pity that concentrations of insulin were not measured at baseline. Second, single-center experience with a limited sample size affects its wide application. Multi-center research and long-term follow-up would allow us to better understand the mechanism of levels IGF-1, IGFBP-1, and IGFBP-1/IGF-1 ratio in identifying HF subtypes and predicting clinical outcomes. Third, we enrolled control groups referred for elective angiography or treatment of uncontrolled hypertension, which may not be representative of the general population. However, this would better reflect real-world clinical scenarios where a diagnosis of HF would be important for guiding management. Finally, metabolic abnormalities especially diabetes mellitus would influence the levels of IGF-1/IGFBP-1. There was a borderline significant difference between the frequency of diabetes in the HF and control groups. Further studies are required to expand on the sample size to allow us to conduct further analyses, such as propensity score matching for diabetes status and HbA1c levels.

## Conclusion

The concentrations of plasma IGFBP-1 and IGFBP-1/IGF-1 ratio can distinguish patients with and without HF. In HF, IGFBP-1 and IGFBP-1/IGF-1 ratio positively correlated with NT-proBNP levels.

## Data Availability Statement

The raw data supporting the conclusions of this article will be made available by the authors, without undue reservation.

## Ethics Statement

The studies involving human participants were reviewed and approved by the Local Ethics Committee of the Second Hospital of Tianjin Medical University. The patients/participants provided their written informed consent to participate in this study.

## Author Contributions

TL and GL put forward conception and study design. SG and MG researched data, tested biomarkers, wrote the manuscript, and contributed to statistical analysis. GT, SG, and TL edited and contributed to the manuscript, data interpretation, and discussion. All authors have read and approved the final version of the manuscript.

## Funding

This study was supported by grants from the Tianjin Natural Science Foundation (Grant Nos. 20JCZDJC00340 and 20JCZXJC00130 to TL) and the National Natural Science Foundation of China (Grant No. 81970270 to TL).

## Conflict of Interest

The authors declare that the research was conducted in the absence of any commercial or financial relationships that could be construed as a potential conflict of interest.

## Publisher's Note

All claims expressed in this article are solely those of the authors and do not necessarily represent those of their affiliated organizations, or those of the publisher, the editors and the reviewers. Any product that may be evaluated in this article, or claim that may be made by its manufacturer, is not guaranteed or endorsed by the publisher.
